# Comparative Analysis of *Artemisia* Plastomes, with Implications for Revealing Phylogenetic Incongruence and Evidence of Hybridization

**DOI:** 10.3390/genes16101145

**Published:** 2025-09-27

**Authors:** Xinqiang Guo, Yonghe Bai, Jing Ruan, Xin Jin, Shang Wang, Dawei Xue, Yuhuan Wu

**Affiliations:** 1 College of Life and Environmental Sciences, Hangzhou Normal University, Hangzhou 311121, China; xqguo@hznu.edu.cn (X.G.); 2023111010068@stu.hznu.edu.cn (Y.B.); jingr627@163.com (J.R.); jxinaaa@163.com (X.J.); wangshang0205@hznu.edu.cn (S.W.); dwxue@hznu.edu.cn (D.X.); 2 Zhejiang Provincial Key Laboratory for Genetic Improvement and Quality Control of Medicinal Plants, Hangzhou Normal University, Hangzhou 311121, China

**Keywords:** *Artemisia*, Asteraceae, chloroplast genome, hybridization, incongruence

## Abstract

**Background**: With the advancement of the next-generation sequencing technology, it is becoming more cost-effective to obtain plastomes from genome skimming data at shallow sequencing depth. *Artemisia* is a species-rich genus, comprising species of great medicinal or economic value. However, plastomes of *Artemisia* have not been thoroughly and comparatively analyzed, and the phylogenetic relationships within the genus are still not well resolved. **Methods**: In this study, 19 *Artemisia* plastomes were obtained from genome skimming data. Together with the plastomes retrieved from the public database, comparative analyses of their structure were also conducted. We further used sequences of plastomes and nuclear internal transcribed spacer sequences to conduct phylogenetic reconstruction. **Results**: The *Artemisia* plastomes are conserved in terms of structure, GC content, gene number, and order. Some regions, i.e., *accD*, *ccsA*, *ndhE*, *ycf1*, *ccsA-ndhD*, *trnG^GCC^-trnfM^CAU^*, were found to be variable and could be chosen as candidates for the DNA barcode. Phylogenetic analyses also confirmed that the four subgenera of *Artemisia* are not monophyletic. The incongruence between plastid and nuclear phylogenies indicated that hybridization events have occurred during the evolution of the genus. **Conclusions**: Reconstructed phylogenies using plastome sequences and nuclear internal transcribed spacers improved our understanding of the phylogenetic backbone of *Artemisia*. In the future, more taxa of *Artemisia* should be sequenced and analyzed to clarify the evolutionary history.

## 1. Introduction

A robust phylogeny is the foundation for understanding the relationships of species and their evolutionary history. Phylogenetic reconstructions are usually difficult due to the intricate evolutionary processes (e.g., hybridization, introgression, and incomplete lineage sorting) [1,2]. In particular, when only a few molecular markers are used or the taxa are incompletely sampled, the phylogenies usually fail to unveil the real relationships [3,4,5].

*Artemisia* L. is a species-rich genus in the family Asteraceae, comprising about 400 species mainly distributed in the Northern Hemisphere, with a few species in the Southern Hemisphere [6,7]. Many species of the genus dominate arid and steppe landscapes, or possess high therapeutic values. Some species are widely used as traditional Chinese medicine [8]. The most famous one is *Artemisia annua* L., from which the anti-malaria artemisinin was extracted [9,10]. Some species are also cultivated as vegetables (e.g., *Artemisia selengensis* Turcz. ex Besser), or used to make alcohol (e.g., *Artemisia dracunculus* L.) [11].

*Artemisia* was considered one of the most taxonomically challenging taxa that has recently undergone rapid evolutionary radiations with complex variation patterns of characters [12,13]. The genus was generally divided into four large infra-generic groups ranked as subgenera, sections, or series based on morphological characters primarily associated with the type of capitula, the fertility of disk floret, and the hairiness of receptacle [13]. Different infra-generic classifications have been consecutively proposed [6,7,14,15,16]. For example, Ling et al. [6] divided the Chinese *Artemisia* into seven sections, including sect. *Abrotanum* (Duhamel) Besser, sect. *Absinthium* (Miller) Candolle, sect. *Albibractea* Y.R. Ling, sect. *Artemisia*, sect. *Dracunculus* Besser, sect. *Latilobus* Y.R. Ling, and sect. *Viscidipubes* Y.R. Ling, and recognized the independent genus *Seriphidium* (Less.) Fourreau. The generally accepted classification includes five subgenera, i.e., subg. *Absinthium* (Miller) Less., subg. *Artemisia*, subg. *Dracunculus* (Besser) Rydb., subg. *Seriphidium* Besser ex Less., and the recently recognized subg. *Tridentatae* (Rydb.) McArthur [7,11,16]. However, in the last three decades, numerous molecular phylogenetic studies have been conducted based on plastid or nuclear DNA fragments (e.g., ITS, *psbA–trnH*, or *trnS–trnC*), and revealed that these subgenera were not monophyletic [15,17,18]. Species previously assigned to subg. *Artemisia* were even scattered across several clades. Using nuclear single-nucleotide polymorphism (SNP) data, Jiao et al. [13] further confirmed the results and found that the morphological characters traditionally used for the infra-generic division did not match the new phylogenetic tree. These inconsistencies also indicated that *Artemisia* experienced a more complex evolutionary history than previously thought.

The chloroplast of angiosperms usually has a structurally conserved circular genome comprising four regions, i.e., the large single-copy (LSC) region, the small single-copy (SSC) region, and the two inverted repeat (IR) regions [19,20,21,22]. The plastome (chloroplast genome) is typically 115–160 kb in length, containing 110–130 genes [23]. It is maternally inherited, evolves at a moderate rate, and also contains sufficient informative sites for phylogenetic construction [24,25,26]. These characters make plastome an ideal marker to resolve phylogenetic relationships with higher resolution, track maternal history, and reveal hybridization events [4,27,28,29,30,31,32]. With the advancement of the next-generation sequencing technology, it is becoming more efficient and cost-effective to obtain and de novo assemble plastomes from genome skimming data at shallow sequencing depth, and an increasing number of plastomes have been published and shared [33]. Up to 31 March 2025, nearly 150 *Artemisia* plastomes are available in NCBI (The National Center for Biotechnology Information; https://www.ncbi.nlm.nih.gov). It thus provides an opportunity for us to conduct a comprehensive phylogenetic analysis of *Artemisia* at the genomic level with more sampling.

Hybridization is recognized as a significant and fundamental biological process in the evolution of animals and plants [31,34,35,36,37,38,39], and it is particularly common in plants [40]. Hybridization processes often lead to the chloroplast transferring from one species to another, resulting in incongruence between nuclear and plastid phylogenies [39,41,42]. Thus, cytonuclear discordance has been a good first approximation for the detection of hybridization events in evolutionary history [43,44,45]. Until now, the plastid and nuclear phylogenies of *Artemisia* have not been thoroughly compared. The occurrence of cytonuclear incongruence and a possible role for hybridization in the evolutionary history of *Artemisia* have not yet been investigated.

In this study, we newly sequenced and assembled 19 plastomes of *Artemisia* using genome skimming data, representing nine species and three varieties. Combined with the plastome data retrieved from the public database, we conducted a comparative analysis of plastomes and constructed plastid phylogenies for this genus. We further constructed nuclear phylogenies using the internal transcribed spacer (ITS) sequences, and compared them with plastid phylogenies to detect the phylogenetic incongruence. Specifically, we also aimed to explore whether hybridization played a role in the evolutionary history of *Artemisia*.

## 2. Materials and Methods

### 2.1. Plant Material, DNA Extraction, and Sequencing

In this study, we newly sequenced 19 samples representing nine species and three varieties (Table 1). The materials were collected in Gansu, Hebei, Inner Mongolia, Qinghai, Shaanxi, Shanxi, Sichuan, Xizang, and Yunnan provinces in China during our field trips in 2016–2021. Fresh leaves were deposited in silica gel for DNA extraction. The voucher specimens were all deposited in the Herbarium of South China Botanical Garden, Chinese Academy of Sciences (IBSC). The detailed information (taxon name, voucher specimen, locality) of these materials is provided in Table S1.

Total DNA was extracted from silica gel-dried leaves using a modified cetyltrimethylammonium bromide (CTAB) method [46] or using the Magnetic Plant Genomic DNA Kit provided by TIANGEN BIOTECH (BEIJING) Co., Ltd. (Beijing, China). The DNA degradation and contamination were detected through electrophoresis on 0.8% agarose gels, and DNA purity was checked using a NanoPhotometer^®^ spectrophotometer (Implen, Calabasas, CA, USA). The library was generated using Truseq Nano DNA HT Sample preparation kit (Illumina, San Diego, CA, USA). Samples were fragmented by sonication, fragments were end-polished, A-tailed, and ligated with the full-length adapter for Illumina sequencing with further polymerase chain reaction (PCR) amplification. PCR products were purified (AMPure XP system, Beckman Coulter, Brea, CA, USA), and libraries were analyzed and quantified. The libraries were sequenced using an Illumina HiSeq 2500 platform, which is more cost-effective, with a paired-end reading length of 150 bp at Novogene Biotechnology Co., Ltd. in Tianjin, China. For each sample, approximately 20 Gb of raw sequencing data at a 10× sequencing depth was generated.

### 2.2. Plastome Assembly and Annotation

The raw sequencing reads were filtered, and the low-quality data located in the joints or ends were removed with Trimmomatic v0.39 [47] using the default parameters. Clean reads were de novo assembled using GetOrganelle v1.7.6.1 [48] and NOVOPlasty v4.3.1 [49] with the plastome sequence of *A. annua* (NCBI accession MG951482.1) as reference. Assembled plastomes were annotated using Plastid Genome Annotator (PGA) [50] with the plastome of *A. annua* (NCBI accession MG951482.1) as reference. The annotation results, especially the start/stop codons and intron/exon boundaries, were manually checked and modified in Geneious Prime v2021 [51]. OGDRAW v1.3.1 [52] was used to visualize the plastome map. The annotated sequences of these plastomes were deposited in GenBank, and the accession numbers are provided in Table 1 and Table S1. We also downloaded 92 published *Artemisia* and three *Ajania* plastomes from NCBI (Table S2). These plastomes were re-annotated using PGA [50] and manually re-checked in Geneious Prime v2021 [51].

### 2.3. Comparison of Plastome Structures, Divergence Analyses, and SSR Search

According to previous studies, the plastomes of *Artemisia* are relatively structurally conservative. In this study, nineteen plastomes representing nine species and three varieties were newly sequenced and assembled, and they were relatively conservative in length of LSC, SSC, and IR regions, GC content, and gene number. We only chose five of them (*A. desertorum*, *A. dracunculus*, *A. eriopoda*, *A. giraldii* var*. longipedunculata*, and *A. tridactyla*), which were previously not or rarely comparatively analyzed and belong to subg. *Dracunculus*, to detect the expansion and contraction of the IR regions among them. The online program IRscope (https://irscope.shinyapps.io/irapp/) (online version accessed on 12 December 2024) [53] was used to visualize the borders of the LSC, SSC, and IR regions.

To detect the variability of different regions of *Artemisia* plastomes, 79 protein-coding sequences (CDS) and 102 inter-generic regions (IGS) of 111 *Artemisia* plastomes (Table S3) were extracted from the GenBank format files using a custom Perl script. We manually checked the CDS and IGS with a low alignment ratio to examine and exclude the potential errors for assembly or annotation in Geneious Prime v2021. Each region was multiple aligned using Muscle5 [54] implemented in Geneious Prime v2021. The degree of variability for each region (CDS and IGS) was quantified by calculating the nucleotide diversity (π) using DnaSP v6 [55].

The MISA-web application (https://webblast.ipk-gatersleben.de/misa/) (accessed on 30 December 2024) [56] was used to detect simple sequence repeat (SSR) of 75 *Artemisia* plastomes, including 19 newly sequenced and 56 previously published (Table S4). A minimum number of repeat units was set as 10 for mononucleotide, five for dinucleotide, four for trinucleotide, and three for tetranucleotide, pentanucleotide, and hexanucleotide. Furthermore, the number and percentage of different types of SSR were analyzed.

### 2.4. Phylogenetic Inference

For phylogenetic reconstructions, we prepared three data matrices in this study, including the concatenated 79 protein-coding sequences (CDS), whole plastome sequences, and the nuclear internal transcribed spacer (ITS) regions. Each CDS was individually aligned using Muscle5 implemented in Geneious Prime v2021. The aligned CDSs were trimmed and concatenated into a single CDS data matrix. The concatenated CDS data matrix and whole plastome data matrix both included 114 samples representing 101 species and 5 varieties, including 3 samples of *Ajania*, i.e., *Ajania fruticulosa* (Ledeb.) Poljak., *Ajania khartensis* (Dunn) Shih., and *Ajania tenuifolia* (Jacq.) Tzvel., which were chosen as outgroups. The ITS data matrix included 83 samples representing 73 species and 3 varieties, including *Ajania fruticulosa* (Ledeb.) Poljak., *Ajania khartensis* (Dunn) Shih., and *Ajania tenuifolia* (Jacq.) Tzvel., which were chosen as outgroups (Table S5). Among them, the ITS sequences of 21 samples were newly generated in this study, and the others were downloaded from GenBank (Table S5). The ITS data matrix was also aligned and trimmed following the same procedure.

The phylogeny was reconstructed using the maximum likelihood method and the Bayesian inference method. The jModelTest v2.1.4 [57] software was used to predict the optimal model for maximum likelihood (ML) analysis. The GTR+G model was predicted as the best model for two datasets, and the node support was evaluated by 1000 bootstrap replicates with 100 random additions per replicate using a fast bootstrapping algorithm. The ML analyses were carried out using RAxML version 8.2.9 [58]. The Bayesian inference (BI) analysis was carried out using MrBayes v3.2 [59,60] with the GTR model. The Markov chain Monte Carlo algorithm was run for 10,000,000 generations, and 1000 generations were used for tree sampling with the first 25% discarded as burn-in to ensure the stable state of each chain. The remaining trees were utilized for building the BI tree with posterior probabilities. The convergence and stationarity of results were checked in Tracer v1.7 [61]. The posterior probabilities (PP) were calculated. Bootstrap percentage (BS) values ≥ 70 [62] and PP values ≥ 0.95 were regarded as strong support. The final phylogenetic trees were viewed in FigTree v1.3.1 [63].

## 3. Results

### 3.1. The General Features of Artemisia Plastomes

**Figure 1:**
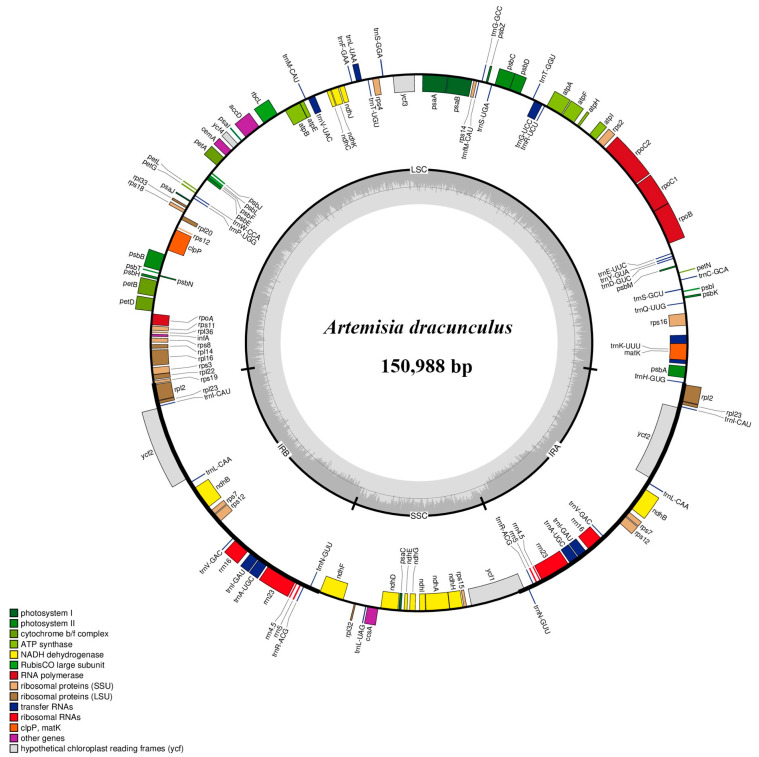
Gene map of the plastome of *Artemisia dracunculus* L. Genes shown inside the circle are transcribed clockwise, and those shown outside are transcribed counter-clockwise. Genes belonging to different functional groups are shown in different colors. The darker gray color in the inner circle corresponds to the GC content, and the lighter gray color corresponds to the AT content. IR, inverted repeat region; LSC, large single copy; SSC, small single copy.

With the Illumina sequencing platform, an average of 20 Gb raw data was produced at a 10× sequencing depth for each sample. All 19 *Artemisia* plastomes newly sequenced in this study display a typical quadripartite structure, consisting of one LSC region (82,535–82,900 bp), one SSC region (18,244–18,325 bp), and two IR regions (24,926–24,963 bp). The total length ranged from 150,654 bp (*A. tridactyla*) to 151,125 bp (*A. desertorum* var. *desertorum*). Total GC contents did not show significant differences, which were 37.4% or 37.5% (Figure 1, Table 1 and Table S1). The gene content and order were also highly conservative, with 132 genes, including 87 protein-coding genes, 37 tRNA genes, and 8 rRNA genes (Table 1, Tables S1 and S2). No gene rearrangement or inversion events were found.

**Figure 2:**
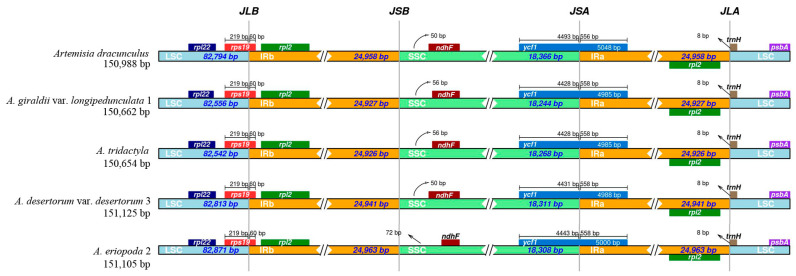
Comparison of the borders of large single-copy (LSC), inverted repeat (IR), and small single-copy (SSC) regions among five *Artemisia* plastomes. JLB (IRb /LSC), JSB (IRb/SSC), JSA (SSC/IRa), and JLA (IRa/LSC) denote the JSs between each corresponding region in the plastomes.

The junctions between the single-copy region and IR regions are conservative among the five plastomes examined in this study (Figure 2). The length of IR regions is relatively consistent and ranges from 24,926 to 24,963 bp. The boundaries of LSC/IRb (JLB) are all crossed by *rps19*. The boundaries of SSC/IRa (JSA) are crossed by the gene *ycf1* with slight variation in length. The junctions of LSC/IRa (JLA) are located within the regions between *rpl2* and *trnH*.

### 3.2. Sequence Divergence, SSR Comparison, and Barcode Identification

**Figure 3:**
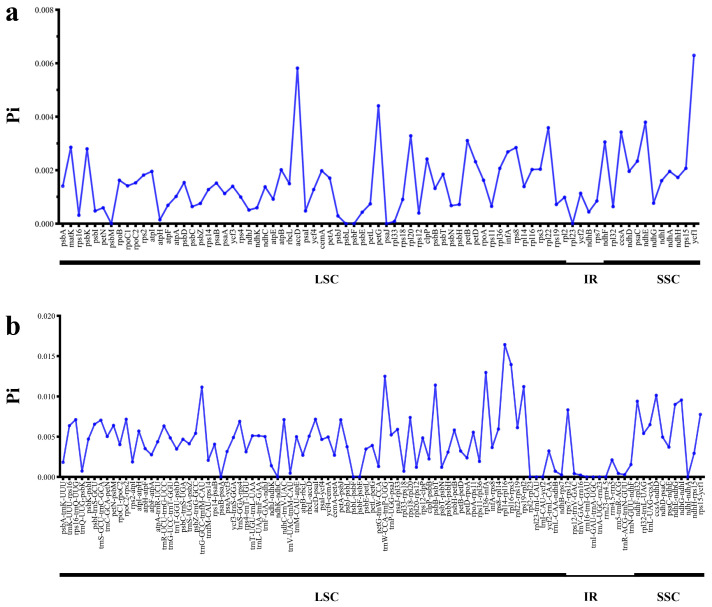
The proportion of parsimony informative sites (Pi) in the 79 *Artemisia* plastomes. (**a**). Protein-coding sequences. (**b**). Intergenic regions. These regions are arranged according to their location in the plastome.

The degree of sequence variability for CDSs and IGSs of *Artemisia* plastomes was detected by calculating the nucleotide diversity (Pi). The values range from 0 to 0.0063 in CDSs, and range from 0 to 0.01645 in IGSs. The IGSs exhibit a higher level of variability compared to the CDSs. Most of the 79 CDSs are rather conserved with Pi values lower than 0.002. Six genes are highly divergent, including *accD*, *ccsA*, *ndhE*, *petG*, *rpl22*, and *ycf1* (Figure 3a and Table S3), and eight IGSs are highly divergent, including *ccsA-ndhD*, *trnG^GCC^-trnfM-^CAU^*, *rps19-rpl2*, *psbB-psbT*, *trnW^CCA^-trnP^UGG^*, *rpl36-infA*, *rpl16-rps3*, and *rpl14-rpl16* (Figure 3b and Table S3). These regions exhibit a high proportion of parsimony-informative sites and were deemed suitable for phylogenetic analysis.

**Figure 4:**
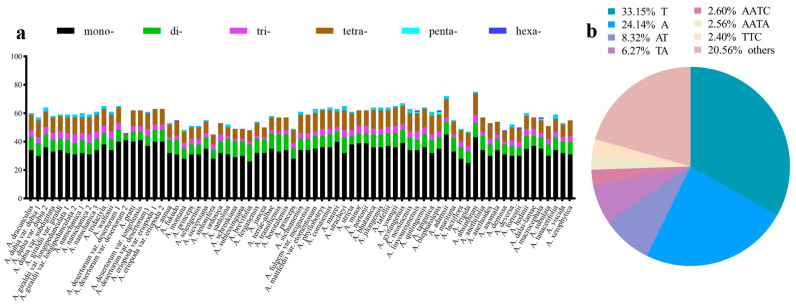
The number of the simple sequence repeats (SSRs) in the 75 *Artemisia* plastomes. (**a**) Number of SSRs in each species. mono-, mononucleotides; di-, dinucleotides; tri-, trinucleotides; tetra-, tetranucleotides; penta-, pentanucleotides; hexa-, hexanucleotides. (**b**) Distribution of different kinds of SSRs.

The number of SSRs in 75 plastomes varies from 45 to 75 (Figure 4 and Table S4). The smallest number of SSRs is found in *A. stolonifera* (45), and the largest is found in *A. absinthium* (75). The number of mononucleotide SSRs is the largest in these plastomes, ranging from 25 (*A. frigida*) to 45 (*A. adamsii*). The dinucleotide SSR number varies from 4 (*A. desertorum* var. *desertorum*) to 12 (*A. brevifolia*, and *A. transiliensis*). Trinucleotide SSR number ranges from 1 (*A. desertorum* var. *desertorum*) to 6 (*A. giraldii* var. *longipedunculata*, *A. nanschanica*, *A. linyoureunensis*, and *A. absinthium*). The number of the tetranucleotide SSR varies from 0 (*A. desertorum* var. *desertorum*) to 15 (*A. absinthium*). The number of pentanucleotide SSRs varies from 0 to 3. Only the plastomes of *A. fukudo*, *A. demissa*, *A. forrestii*, *A. linyoureunensis, A. macrocephala*, and *A. blepharolepis* have one hexanucleotide SSR.

A total of 4341 SSRs were found. Seven classes of SSRs are highly frequent (Figure 4 and Table S4). Poly (T) and (A) repeats account for 57.29% of the total SSRs. Dinucleotides (14.59%) are the next abundant repeat with frequent occurrence of repeat motifs AT (8.32%) and TA (6.27%). Trinucleotides (6.74%) consist of seven repeat motifs; the most frequent is TTC (2.40%). The frequencies of the tetranucleotide and pentanucleotide SSRs are 17.85% and 1.89%, respectively. The AT/TA and AAAG/CTTT are the most frequent units, respectively. There are only six hexanucleotide SSRs accounting for 0.15% of the total SSRs.

### 3.3. Phylogenetic Analyses

**Figure 5:**
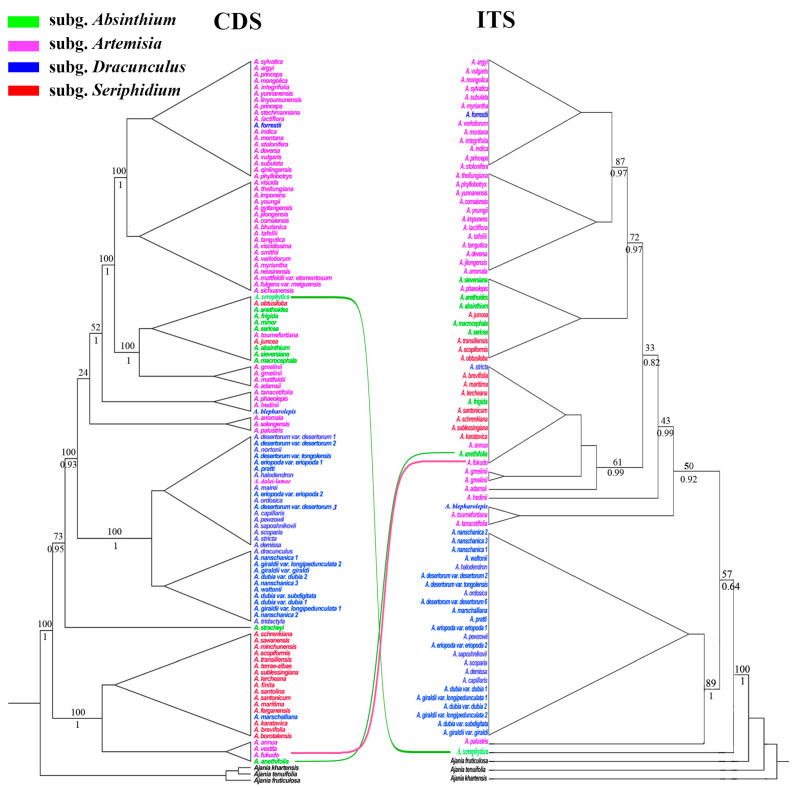
Phylogenetic trees of *Artemisia* based on the protein-coding sequences (CDS) of plastome and nuclear internal transcribed spacer (ITS) sequences (right) using maximum likelihood (ML) methods. Lines indicate the incongruence between the two trees. The numbers above and under the branches are ML bootstrap value (BS) and BI posterior probabilities (PP).

The Maximum likelihood (ML) and Bayesian inference (BI) analyses of each dataset yield similar topologies. Only the ML trees inferred are presented in Figure 5. The topology incongruences were obtained between the plastid and nuclear markers (Figure 5). The phylogenetic trees generated using CDS or nrITS are provided in Figures S1–S4 with ML bootstrap support (BS) or BI posterior probability (PP) values added. The phylogenetic analyses of CDS revealed 11 consecutive primary clades along the backbone of *Artemisia*. Most sampled species of subg. *Seriphidium*, except *A. juncea* Kar. & Kir. and *A. obtusiloba* Ledeb., together with *A. fukudo* Makino, *A. annua* L., and *A. vestita* Bess. of subg. *Artemisia*, and *A. anethifolia* Stechm. of subg. *Absinthium*, formed the basal diverging clade. *Artemisia stracheyi* C. B. Clarke formed an independent clade. All species of subg. *Dracunculus*, except *A. blepharolepis* Bunge, *A. marschalliana* Spreng., and *A. forrestii* W. W. Smith, grouped into a monophyletic clade with *A. dalai-lamae* Krasch. of subg. *Artemisia*. Most samples of subg. *Absinthium*, including *A. absinthium* L., *A. anethoides* Mattf., *A. frigida* Willd., *A. macrocephala* Bess., *A. minor* Bess., *A. obtusiloba* Ledeb., *A. sericea* Stechm, *A. sieversiana* Willd., and *A. xerophytica* Krasch. clustered together into one clade, which was further inserted into a larger clade with species of subg. *Artemisia*. The phylogenetic analyses using ITS revealed 10 consecutive primary clades along the backbone. But the basal diverging clade was formed only by *A. xerophytica*. Most species of subg. *Dracunculus* and *A. palustris* L. of subg. *Artemisia* were grouped into the next basal diverging clade. Most species of subg. *Absinthium* and subg. *Seriphidium* clustered together into two main clades. Consistent with previous phylogenetic studies, none of the four main subgenera (i.e., subg. *Absinthium*, subg. *Artemisia*, subg. *Dracunculus*, and subg. *Seriphidium*) is monophyletic. Species of subg. *Artemisia* and subg. *Dracunculus* were scattered across several clades.

## 4. Discussion

In this study, we newly sequenced and assembled nineteen complete plastomes representing nine species and three varieties. Combined with the plastomes downloaded from the public database, we conducted a thorough comparative analysis of their structure and characteristics. As previously revealed, the *Artemisia* plastomes are typically structurally quadripartite, comprising one LSC, one SSC, and two IR regions [64]. No structural change, such as gene loss and rearrangement, was detected. The plastome size also falls within the variation range of Angiosperm [23]. These results demonstrated the conservative character of *Artemisia* plastomes, which is common in Angiosperms [20]. In fact, stabilizing the gene structure is critical for the plastome [65]. And the contractions or expansions at the boundaries of the IR regions are widely recognized as the primary processes leading to genome size variation. One reason for the conservation of the IR regions is their crucial role in ensuring the structural stability of the plastome [66,67,68]. Our results indicate that the IR region of *Artemisia* is very similar in length and structurally stable. Comparisons with plastomes of related taxa, including genera in the tribe Anthemideae or family Asteraceae, are also needed to fully understand the effect of plastome structural variation in the evolution of *Artemisia*.

From the analysis, we found that some regions contain a high proportion of parsimony informative sites, including genes *accD*, *ccsA*, *ndhE*, *petG*, *rpl22*, and *ycf1*, and intergenic regions *ccsA-ndhD*, *trnGGCC-trnfM-CAU*, *rps19-rpl2*, *psbB-psbT*, *trnWCCA-trnPUGG*, *rpl36-infA*, *rpl16-rps3*, and *rpl14-rpl16* (Figure 3 and Table S3). Some of these regions have been reported in previous analyses. For example, the *rbcL* gene is widely recommended and used as a DNA barcode for species delimitation and phylogenetic construction [69,70,71]. Kim et al. comparatively analyzed 30 plastomes of *Artemisia* in East Asia, and identified *accD*, *rpl32*-*trnL*^UAG^, and *ycf1* as nucleotide diversity hotspots [72]. They revealed that these regions have the potential as candidate regions for the development of universal barcode markers. The genus *Artemisia* is notoriously taxonomically difficult, and misidentifications are rather common, especially in some species complexes [73,74,75]. These regions are potentially useful as molecular markers to improve identification quality and species delimitation. Our research further found that the LSC region has a higher proportion of parsimony informative sites than the IR and SSC regions. Repeat sequences play an important role in genomic rearrangement and structural stabilization of plastomes [76,77,78]. Such repeats are also important in understanding phylogeny and evolutionary history [78]. In this study, a total of 4341 SSRs were identified from the 75 *Artemisia* plastomes (Figure 2 and Table S4). Owing to their characteristics of abundance, maternal inheritance, and haploid nature, plastome SSRs have been used for the analysis of population genetic variation and gene flow [79,80], and are usually considered to be informative markers. The application and significance of SSR markers in other angiosperms have been frequently reported [81]. In the plastomes analyzed here, *A. absinthium* exhibits the highest number of SSRs (75), and *A. keiskeana* has the fewest (43). We further analyzed the types of SSRs in *Artemisia* plastomes. Consistent with the most recent results [65], we also determine that the mononucleotide-type SSRs are predominant, with a bias toward A/T nucleotides, which corresponds with an A/T-rich plastome.

In this study, the tree topology generated by the concatenated CDS of plastomes and nrITS improved our understanding of the relationships within *Artemisia*. Consistent with previous studies, the traditional division of *Artemisia,* mainly based on morphological characters, i.e., type of capitula, fertility of disk floret, and hairiness of receptacle, is not natural. All four subgenera, including subg. *Absinthium*, subg. *Artemisia*, subg. *Dracunculus*, and subg. *Seriphidium*, are not monophyletic. For example, species of subg. *Artemisia* and subg. *Dracunculus* are scattered across different clades. However, many species of the genus are still not sampled; further studies involving more species and combining more evidence (e.g., cytology, morphology, population genetics) will be needed to re-classify the genus. Nevertheless, our results revealed the advantage of using plastome data with more informative sites in resolving phylogenetic relationships. Additionally, it should be noted that the nuclear ITS trees topologically differed from the plastid trees, primarily in relation to the basal diverging clade. The basal diverging clade mainly consisted of species of subg. *Seriphidium* in plastid CDS phylogenies, but included *A. xerophytica* (subg. *Absinthium*) and species of subg. *Dracunsulus* in ITS phylogenies. Conflicts of the position of some species were also discovered, including *A. forrestii*, *A. blepharolepis*, and *A. tournefortiana* Rchb.

It has been revealed that the inconsistent phylogenetic relationships within plants are common if they are constructed based on different regions, especially plastid and nuclear sequences [29,82,83,84,85]. The incongruence can be caused by many different biological factors, including gene recombination, hybridization/introgression, incomplete lineage sorting, horizontal gene transfer, and natural selection [86,87,88]. Among these, the most common factors in plants are hybridization/introgression and incomplete lineage sorting [87,88,89]. Although not rigorously tested in this study, our current knowledge of this group suggests that both processes may have played a significant role in the evolution of *Artemisia*. First, hybridization/introgression is particularly plausible, given that many species are widely and sympatrically distributed, and almost exclusively wind-pollinated [89], which facilitates crosspollination. Indeed, many natural hybrids in this genus have been confirmed through detailed studies [90,91]. Moreover, intergeneric hybrids between *Chrysanthemum* and *Artemisia* are also reported [92,93], indicating that the reproductive barriers for *Artemisia* species are relatively weak. This is also supported by our own observations; during our extensive fieldwork in China, we frequently encountered individuals and populations with intermediate morphology and continuous variation between two well-defined *Artemisia* species. Incomplete lineage sorting is also likely to have substantially shaped the evolutionary history of *Artemisia*. In the phylogenetic backbone of *Artemisia*, two deep nodes within subg. *Artemisia* are generally not well-supported and are associated with very short branches (Figure 5, Figures S1–S4). In addition, the subg. *Artemisia* exhibits the greatest diversity within the genus, characterized by complex morphological patterns and a broad distribution range. This pattern strongly suggests that a rapid radiation event likely occurred during the early divergence of subg. *Artemisia*, with incomplete lineage sorting playing a major role in its evolutionary history. Addressing this unresolved issue will also be a key focus of future studies. Compared to the large size of *Artemisia*, however, the samples in our study are still not enough. It is worth mentioning that the phylogenetic incongruence observed in this study is based on plastid and nrITS data. However, given nrITS’s well-known concerted evolution, it is not an ideal marker for investigating hybridization events. Single- and low-copy nuclear (SLCN) genes, which exhibit biparental inheritance and lack significant concerted evolution, are considered more suitable for such analyses [94,95]. In the future, utilizing the Angiosperm 353 nuclear gene set to further investigate hybridization in this genus is still needed.

## 5. Conclusions

In this study, we sequenced and assembled 19 complete plastomes of *Artemisia*. Combined with previously published plastome data, a thorough comparative analysis between them was conducted. The results indicated that the plastomes of Artemisia are highly conserved. Moreover, some regions containing a high proportion of parsimony informative sites were detected throughout the plastome. Importantly, we reconstructed the most comprehensive plastid phylogeny of *Artemisia* to date, based on 111 plastomes. The results corroborate several key findings from previous studies and also highlight the need for a thorough revision of the infrageneric classification. In combination with the ITS phylogeny, we further revealed the extent of cytonuclear incongruence within this genus, underscoring its complex evolutionary history. Based on current evidence, we infer that both hybridization/introgression and incomplete lineage sorting may have played significant roles in shaping the diversification of *Artemisia*. Collectively, these findings have substantially advanced our knowledge of this group and provide a solid phylogenetic framework for future research. Moving forward, we aim to employ single-/low-copy nuclear genes to more fully resolve the complex evolution of *Artemisia*.

## Figures and Tables

**Table 1:** The statistics summary of 19 newly sequenced plastomes of *Artemisia.*

Taxon	Genbank Accession Number	Length (bp)	GC Content	LSC Size (bp)	IR Size (bp)	SSC Size (bp)	Protein Encoding Gene	tRNA	rRNA
*A. dracunculus*	PQ850015	150,988	0.375	82,773	24,959	18,297	87	37	8
*A. dubia* var*. dubia* 1	PQ850014	151,013	0.375	82,776	24,959	18,319	87	37	8
*A. dubia* var. *dubia* 2	PQ850013	151,024	0.375	82,817	24,959	18,289	87	37	8
*A. dubia* var. *subdigitata*	PQ850012	151,032	0.375	82,794	24,959	18,320	87	37	8
*A. giraldii* var. *giraldii*	PQ850011	151,045	0.375	82,802	24,959	18,325	87	37	8
*A. giraldii* var. *longipedunculata*1	PQ850010	150,662	0.375	82,542	24,926	18,268	87	37	8
*A. giraldii* var. *longipedunculata*2	PQ850009	151,009	0.375	82,783	24,959	18,308	87	37	8
*A. nanschanica* 1	PQ850008	151,008	0.375	82,777	24,959	18,313	87	37	8
*A. nanschanica* 2	PQ850007	150,655	0.375	82,535	24,926	18,268	87	37	8
*A. nanschanica* 3	PQ850006	151,031	0.375	82,804	24,959	18,309	87	37	8
*A. tridactyla*	PQ850005	150,654	0.375	82,556	24,927	18,244	87	37	8
*A. waltonii*	PQ850004	151,042	0.375	82,814	24,959	18,310	87	37	8
*A. desertorum* var*. desertorum* 1	PQ850003	151,014	0.375	82,812	24,959	18,284	87	37	8
*A. desertorum* var*. desertorum* 2	PQ850002	151,102	0.375	82,878	24,959	18,306	87	37	8
*A. pratti*	PQ850001	151,052	0.375	82,847	24,959	18,287	87	37	8
*A. desertorum* var*. tongolensis*	PQ850019	151,061	0.375	82,855	24,959	18,288	87	37	8
*A. desertorum* var*. desertorum* 3	PQ850018	151,125	0.375	82,900	24,963	18,299	87	37	8
*A. eriopoda* 1	PQ850017	151,064	0.375	82,853	24,959	18,293	87	37	8
*A. eriopoda* 2	PQ850016	151,105	0.375	82,871	24,963	18,308	87	37	8

Notes: The “1, 2, 3” after the taxon names represent different samples of the same taxon.

## Data Availability

The original contributions presented in this study are included in the article/Supplementary Materials. Further inquiries can be directed to the corresponding author.

## Supplementary Materials

The following supporting information can be downloaded at: https://www.mdpi.com/article/10.3390/genes16101145/s1. Figure S1: Maximum likelihood phylogenetic tree obtained from protein-coding genes (CDS) of plastomes. The numbers above the branches represent ML bootstrap support (BS) values. Figure S2: Bayesian inference (BI) tree obtained from protein-coding genes (CDS) of plastomes. The numbers above the branches represent Bayesian posterior probability (PP) values. Figure S3: Maximum likelihood phylogenetic tree obtained from nuclear internal transcribed spacers (ITS). The numbers above the branches represent ML bootstrap support (BS) values. The colors of branches indicate the traditional subgeneric classification of *Artemisia*. Figure S4: Bayesian inference (BI) tree obtained from nuclear internal transcribed spacers (ITS). The numbers above the branches represent Bayesian posterior probilities (PP) values. Figure S5: Bayesian inference (BI) tree obtained from whole plastome sequences. The numbers above the branches represent Bayesian posterior probability (PP) values. Figure S6: Maximum likelihood phylogenetic tree obtained from whole plastome sequences. The numbers above the branches represent ML bootstrap support (BS) values. Table S1: The detailed information on the materials of newly sequenced plastomes. Table S2: Information on *Artemisia* and *Ajania* plastomes downloaded from NCBI. Table S3. The nucleotide variability (Pi) values of protein-coding regions (CDS) and intergenic spacer regions (IGS) in *Artemisia*. Table S4: The number of different types of SSRs. Table S5: The detailed information of ITS sequences.

## Abbreviations

CDS	Protein-coding sequences
IGS	Intergenic spacer region
ITS	Internal transcribed spacer
