# Supplementary material for: Comparative Analysis of Artemisia Plastomes, with Implications for Revealing Phylogenetic Incongruence and Evidence of Hybridization
Source: Genes (Basel). 2025 Sep 27;16(10):1145. doi: 10.3390/genes16101145 (PMC12564433; doi:10.3390/genes16101145)
Supplement: Supplementary file 1 [file genes-16-01145-s001.zip › Figure S1-S6.pdf]

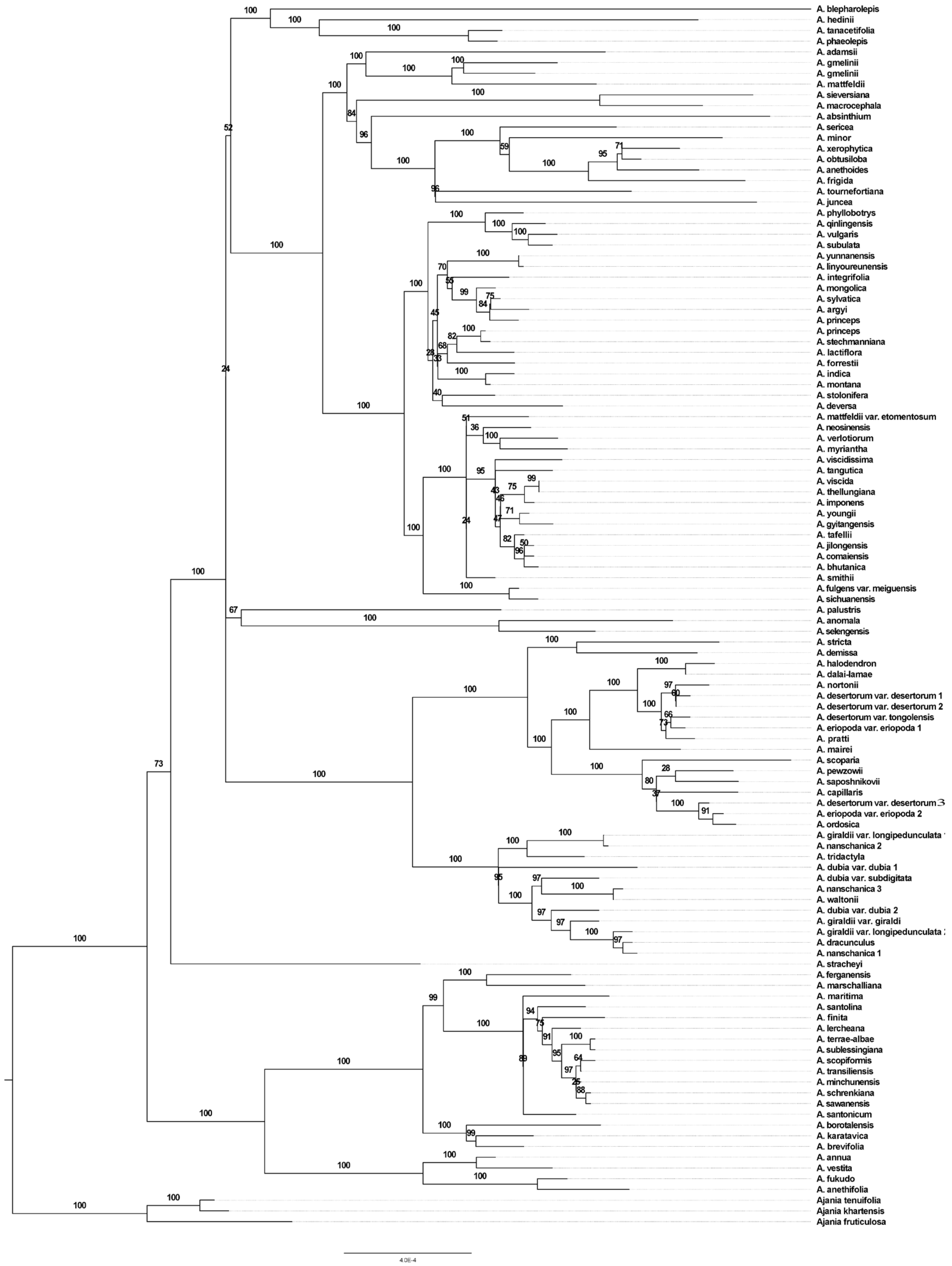

Fig. S1 Maximum likelihood phylogenetic tree obtained from protein coding genes (CDS) of plastomes. The numbers above the branches represent ML bootstrap support (BS) values.

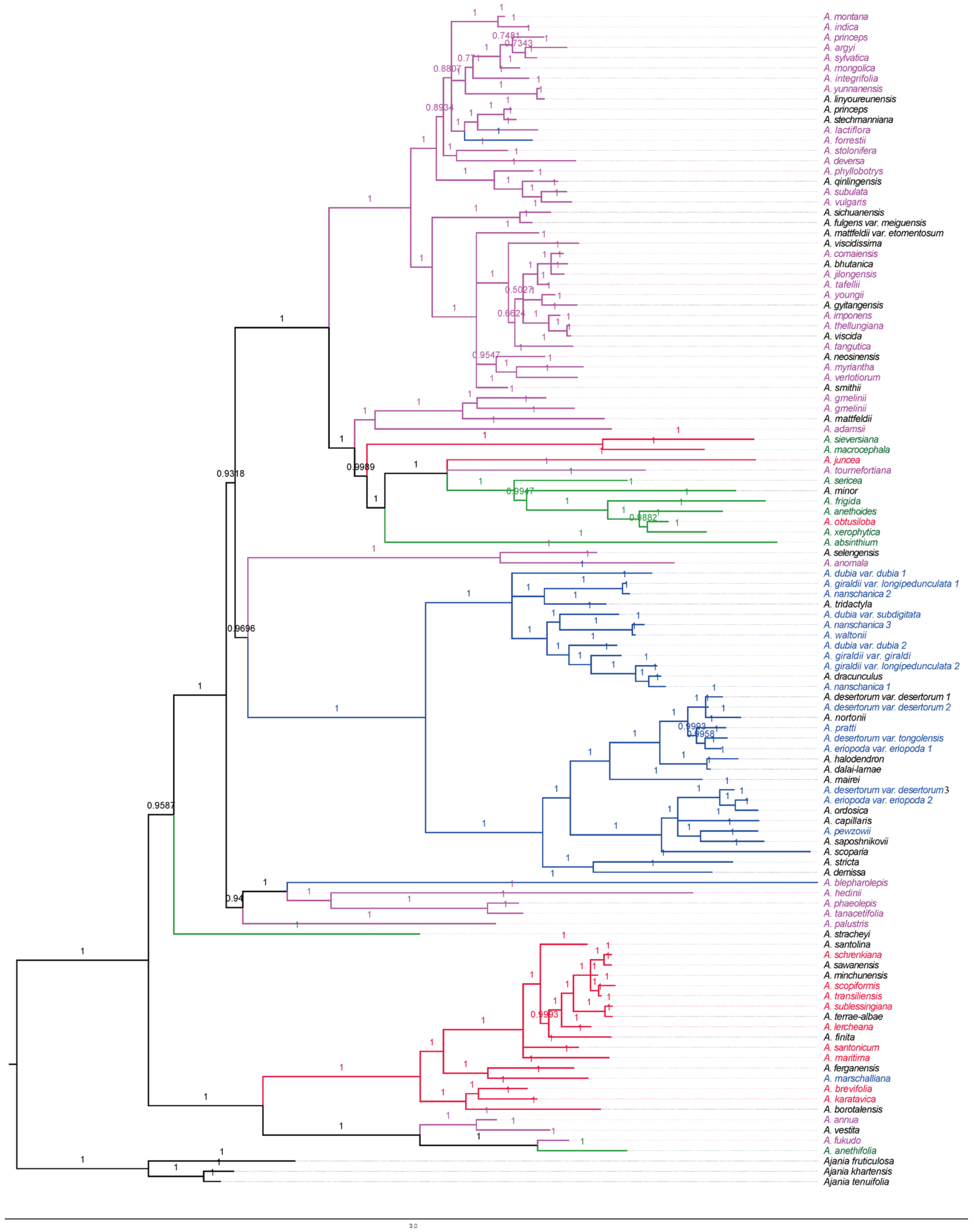

Fig. S2 Bayesian inference (BI) tree obtained from protein coding genes (CDS) of plastomes. The numbers above the branches represent Bayesian posterior probilities (PP) values.

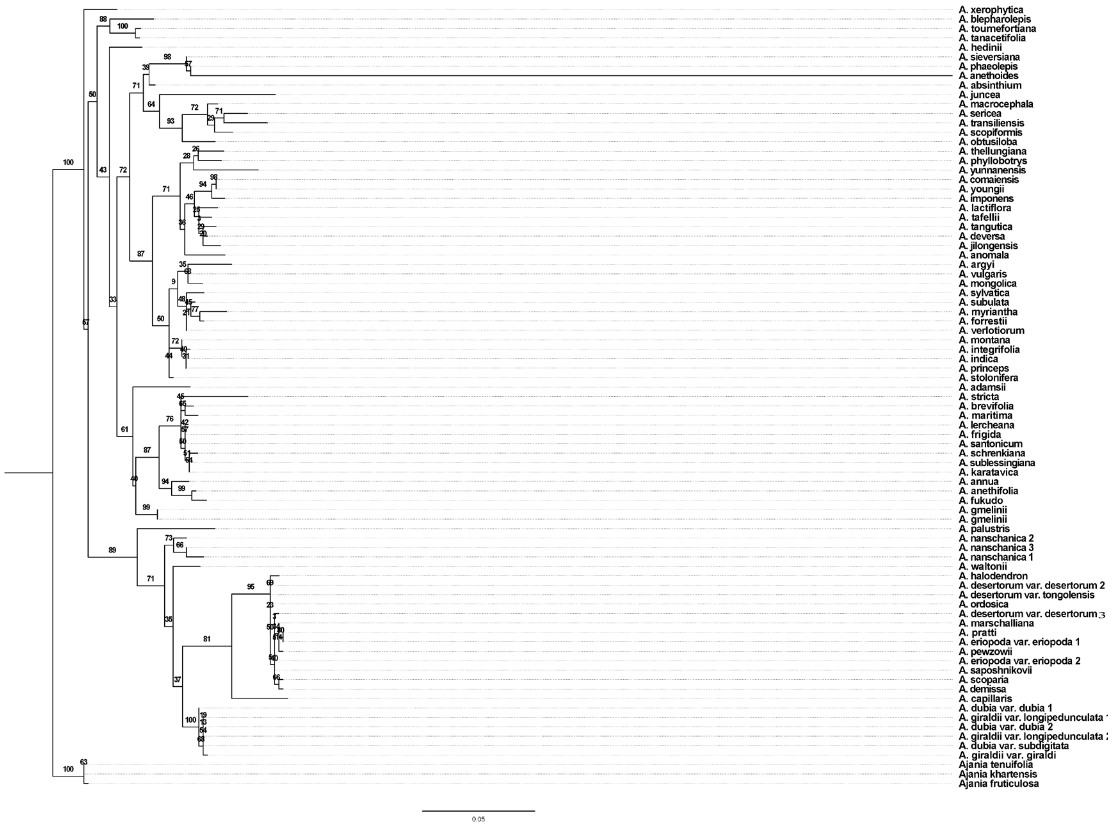

Fig. S3 Maximum likelihood phylogenetic tree obtained from nuclear internal transcribed spacers (ITS). The numbers above the branches represent ML bootstrap support (BS) values.

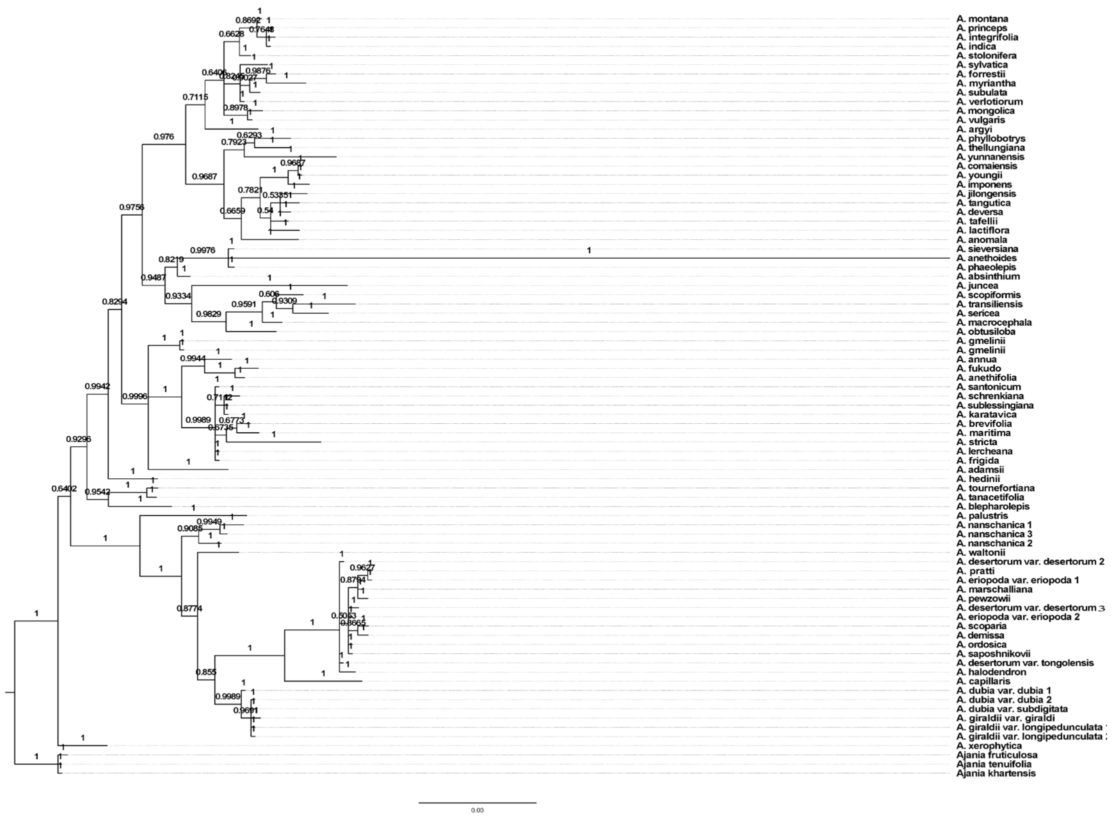

Fig. S4 Bayesian inference (BI) tree obtained from nuclear internal transcribed spacers (ITS). The numbers above the branches represent Bayesian posterior probilities (PP) values. The colors of branches indicate the traditional subgeneric classification of *Artemisia*.

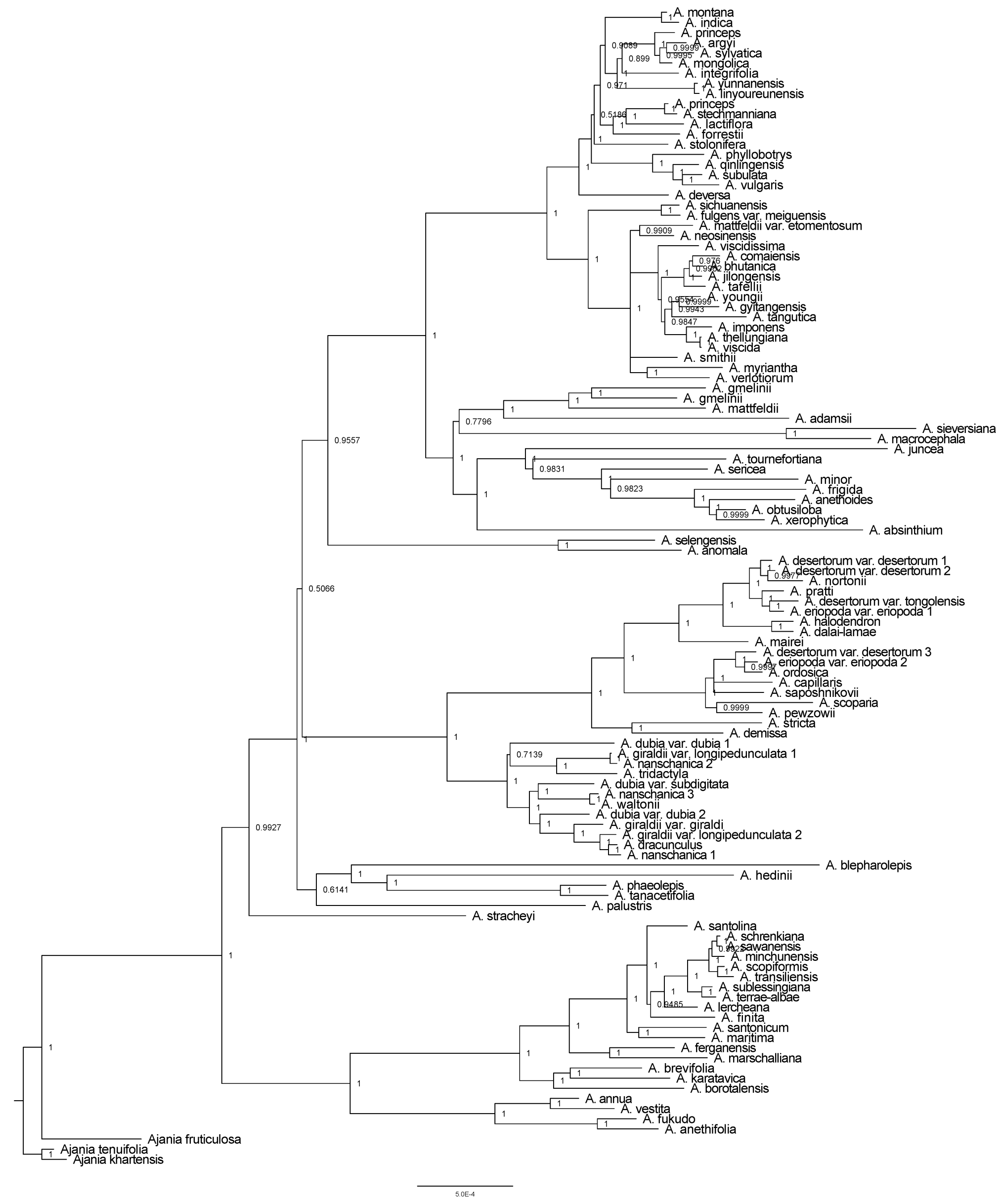

Fig. S5 Bayesian inference (BI) tree obtained from whole plastome sequences. The numbers above the branches represent Bayesian posterior probilities (PP) values.

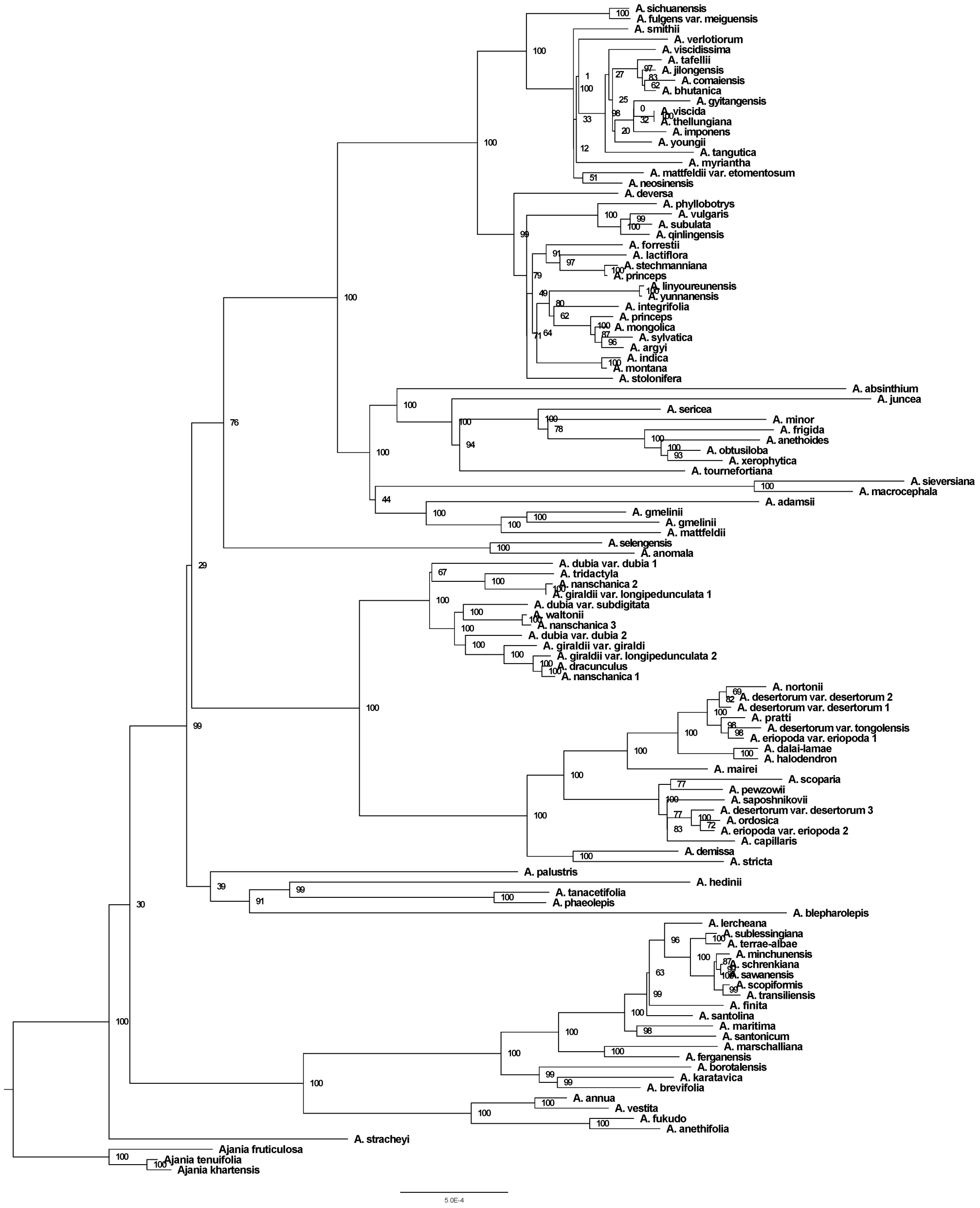

Fig. S6 Maximum likelihood phylogenetic tree obtained from whole plastome sequences. The numbers above the branches represent ML bootstrap support (BS) values.
